# Supplementary material for: Lipid metabolic features of T cells in the Tumor Microenvironment
Source: Lipids Health Dis. 2022 Oct 6;21:94. doi: 10.1186/s12944-022-01705-y (PMC9535888; doi:10.1186/s12944-022-01705-y)
Supplement: Supplementary file 1 — Supplementary Material 1 [file 12944_2022_1705_MOESM1_ESM.pdf]

Certificate of English Language Editing

Manuscript Title:

Lipid Metabolic Features of T cells in the Tumor Microenvironment

Date of Revision:

September 20, 2022

Abstract:

The tumor microenvironment (TME) is characterized by discrete changes in metabolic features of cancer and immune cells, with various implications. Cancer cells take up most of the available glucose to support their growth, thereby leaving immune cells with insufficient nutrients to expand. In the relative absence of glucose, T cells switch the metabolic program to lipid-based sources, which is pivotal to T-cell differentiation and activation in nutrient-stressed TME. Although consumption of lipids should provide an alternative energy source to starving T cells, a literature survey has revealed that it may not necessarily lead to antitumor responses. Different subtypes of T cells behave differently in various lipid overload states, which widely depends upon the kind of free fatty acids (FFA) engulfed. Key lipid metabolic genes provide cytotoxic T cells with necessary nutrients for proliferation in the absence of glucose, thereby favoring antitumor immunity, but the same genes cause immune evasion in Tmem and Treg. This review aims to detail the complexity of differential lipid metabolism in distinct subtypes of T cells that drive the...

This document certifies that the manuscript listed above was copy edited for English language by LetPub, with regard to grammar, punctuation, spelling, and clarity. All of our language editors are native English speakers with long-term experience in editing scientific and technical manuscripts. We are committed to leveling the playing field for researchers whose native language is not English.

- Documents receiving this certification should be regarded as having undergone professional editorial revision for English language before submission. However, the authors may accept or reject LetPub's suggestions and changes at their own discretion and LetPub does not have editorial control over the submitted documents.
- The language quality of the submitted document is the sole responsibility of the submitting authors subject to those authors' adherence to LetPub's revisions and instruction. LetPub's provision of service does not constitute a guarantee or endorsement of the authors' work herein.
- Neither the research content nor the authors' intended meaning were altered in any way during the editing process.
- If you have any questions or concerns about this edited document, please contact us at support@letpub.com

LetPub
Your Manuscript Editor

LetPub is an author service brand owned and operated by Accdon LLC. Headquartered in the Boston area, we are a full-spectrum author services company with a large team of US-based certified language and scientific editors, ISO 17001 accredited translators, and professional scientific illustrators and animators. We advocate ethical publication practices and are an official member of the Committee on Publication Ethics (COPE).

For more information about our company, services, and partnership programs, please visit www.letpub.com.

© 2022 Accdon, LLC. All Rights Reserved. Tel: 1-781-202-9968 Email: info@accdon.com Address: 400 Fifth Ave, Suite 530, Waltham, MA 02451, United States
