# Supplementary figures and images for: Lipid metabolic features of T cells in the Tumor Microenvironment
Source: Lipids Health Dis. 2022 Oct 6;21:94. doi: 10.1186/s12944-022-01705-y (PMC9535888; doi:10.1186/s12944-022-01705-y)

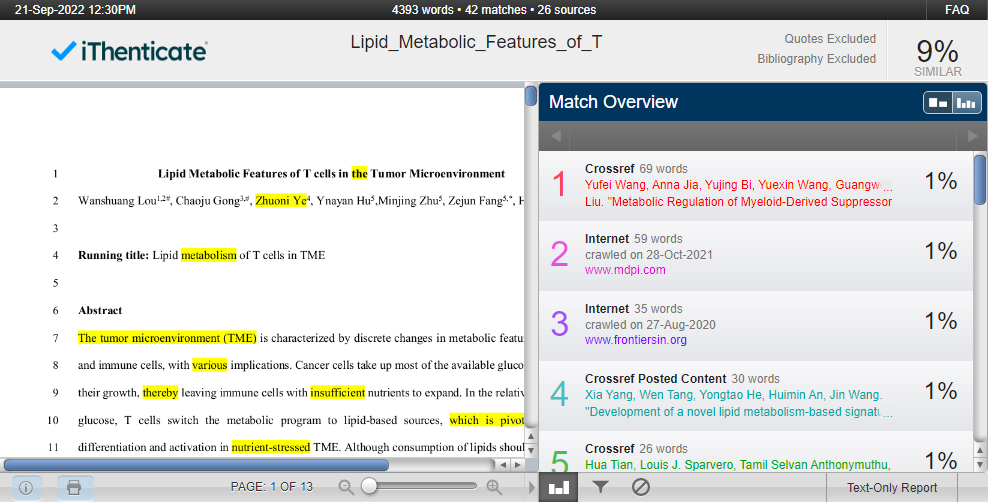

Supplement: Supplementary file 2 — Supplementary Material 2 [file 12944_2022_1705_MOESM2_ESM.png]
