# Supplementary material for: Lipid metabolic features of T cells in the Tumor Microenvironment
Source: Lipids Health Dis. 2022 Oct 6;21:94. doi: 10.1186/s12944-022-01705-y (PMC9535888; doi:10.1186/s12944-022-01705-y)
Supplement: Supplementary file 3 — Supplementary Material 3 [file 12944_2022_1705_MOESM3_ESM.pdf]

Lipid_Metabolic_Features_of_T

WORD COUNT 4393
TIME SUBMITTED 21-SEP-2022 12:30PM
PAPER ID 90727419

Lipid Metabolic Features of T cells in the Tumor Microenvironment

Wanshuang Lou[1,2#], Chaoju Gong[3,#], Zhuoni Ye[4], Ynayan Hu[5],Minjing Zhu[5], Zejun Fang[5,*], Huihui Xu[6,*]

Running title: Lipid metabolism of T cells in TME

Abstract

The tumor microenvironment (TME) is characterized by discrete changes in metabolic features of cancer and immune cells, with various implications. Cancer cells take up most of the available glucose to support their growth, thereby leaving immune cells with insufficient nutrients to expand. In the relative absence of glucose, T cells switch the metabolic program to lipid-based sources, which is pivotal to T-cell differentiation and activation in nutrient-stressed TME. Although consumption of lipids should provide an alternative energy source to starving T cells, a literature survey has revealed that it may not necessarily lead to antitumor responses. Different subtypes of T cells behave differently in various lipid overload states, which widely depends upon the kind of free fatty acids (FFA) engulfed. Key lipid metabolic genes provide cytotoxic T cells with necessary nutrients for proliferation in the absence of glucose, thereby favoring antitumor immunity, but the same genes cause immune evasion in T_{mem} and T_{reg}. This review aims to detail the complexity of differential lipid metabolism in distinct subtypes of T cells that drive the antitumor or pro-tumor immunity in specific TME states. We have identified key drug targets related to lipid metabolic rewiring in TME.

Keywords: Lipid; T-cells; tumor microenvironment (TME); PD-L1/2; CD36

Introduction

Our bodies utilize lipids for various fundamental processes, ranging from the major energy source to the synthesis of vital macromolecules such as cholesterol, membrane phospholipids, and hormones. Like most cells, T cells are heavily dependent on lipid consumption for energy needs, but their naiveté, activation, and effector functions are influenced by the subtype and quantity of lipid intake [1]. A number

of surface and intracellular proteins, such as differentiation 36 (CD36), fatty acid-binding protein (FABP), fatty acid transporter protein (FATP), and sterol regulatory-element binding proteins (SREBPs), are responsible for processing lipids in T cells. Under normal circumstances, quiescent T cells process lipids into more energy-efficient oxidative phosphorylation (OXPHOS), which is replaced by aerobic glycolysis once T cells are activated. A complex cascade of co-stimulatory triggers channel T cells into T-regulatory cells (T_{regs}) and helper T cells (Th cells), each with differential metabolic shifts. As the immune response approaches an end, activated T cells undergo apoptosis or are converted to nondividing T memory cells that revert to OXPHOS [2, 3].

The tumor microenvironment (TME) is a complex space characterized by multiple cell types and their interwoven interactions, predominantly favoring cancerous growth. The interdependence of nutrients, vasculature, and metabolic demands actively shapes the cellular fate in TME. The specialized metabolic switches, nutrient preferences, cellular growth demands, and secretion of various intrinsic and extrinsic factors prime the particular kind of cell in TME to behave in a specific manner [4, 5]. For example, growth signals and upregulation of metabolic features in tumor cells make them more suitable for proliferation, while immune cells undergo tumor evasion after metabolic switch at the same time [6, 7]. As proliferating cells, in contrast to normal cells, require macromolecule biosynthesis and redox homeostasis in addition to their normal energy needs [8-10], rapidly growing cancerous cells overtake metabolic control in TME, resulting in compromised nutrients supply to immune system cells [11]. In fact, the effector functions of innate and adaptive cells are partially controlled by their ability to consume glucose, which is mediated by glucose transporter-1 (GLUT1) receptors [12]. A glycolytic challenge within TME curbs the effector function of immune cells on one side while enabling tumor cells with additional glucose to thrive on the other. Beyond the evident impact of glucose metabolism on immune cell reprogramming in TME, the role of lipid metabolism remains relatively poorly studied despite its conceivable implications.

Cancer typically withstands nutrient deprivation by interacting with nearby stromal cells. Cancer cells activate neighboring adipocytes to provide a sustained supply of lipids for tumor survival and

proliferation [13, 14]. Another important source of lipids in TME are cancer-associated fibroblasts [15, 16]. Moreover, cancer cells reprogramming to initiate de novo lipid synthesis, upregulation of fatty acid binding, and uptake of proteins ensures the surplus energy source in TME. Preferential accumulation of lipids in TME, greater availability of fatty acids to cancer cells, and subsequent immune evasion are the hallmarks of the lipid metabolism features in TME. Although the metabolic narrative of cancer is increasingly accepted, scattered data remain a challenge. This review aims to identify and highlight the lipid metabolic features of T cells in TME.

Genes involved in fatty acid-driven differentiation of T cells in TME

Activated T cells have greater metabolic demands to assist their proliferation, which requires higher de novo fatty acid synthesis (FAS), manifested by the conversion of glucose to fatty acids [17]. Previous studies have revealed that sterol-responsive element-binding proteins (SREBPs) induce FAS in activated T cells via mTORC1 [18-20], while naïve T cells and memory T cells (T_{mem}) maintain fatty acid oxidation (FAO) as the default metabolic program. FAS has to go through various steps mediated by key enzymes before lipid synthesis can occur. Some of these enzymes affect the rewiring of T cells. For instance, it has been found that $CD8^+$ T cells cannot expand without SREBP signaling during viral infection; however, it was expendable for homeostatic growth. SREBPs in T_{effs} induce the expression of enzymes fatty acid synthetase (FASN), acetyl-CoA carboxylase (ACC) and hydroxy-methyl-glutaryl-CoA reductase (HMGCR) [17]. Seon Ah Lim et al. have reinforced these observations in a mouse model of TME, showing that inhibition of SREBP-dependent lipid synthesis and metabolic reprogramming in T_{regs} initiates a robust antitumor response without causing autoimmune disorders. It has been further proven that depletion of an obligatory SREBP factor SCAP curtails tumor growth and upgrades anti-PD-1 immunotherapy [21]. Similarly, Yang-An Wen et al. confirmed the tumor growth suppression after the knockdown of either SREBP1 or SREBP2 target genes required for lipid biosynthesis [22]. At least theoretically, depletion of SREBPs in cancer cells and T_{regs} yields anticancer benefits; however, deletion of SREBPs in T_{effs} can affect their blast, which may lead to immune evasion. These SREBPs might be

context-dependent drug targets. Further research is required to ascertain them in all subsets of T cells and cancer cells in complex TME.

In addition to SREBPs, ACC is another widely studied lipogenic gene in this direction, which has led to similar results. For instance, Luciana Berod and colleagues found that TH17 cells, but not T_{regs}, rely on the de novo lipid synthesis mediated by ACC1. T_{regs} do not follow this scheme as they tend to utilize exogenous FFAs. T cell–specific deletion of ACC1 in mice is able to ameliorate autoimmune disease through anti-inflammatory actions [23]. Similarly, CD8+ T-cell expansion and proliferation are severely curtailed in the absence of ACC1 and subsequently restored by exogenous fatty acid supply [24]. Inhibition and activation of ACC1 favor peripheral T_{regs} and Th17 cell differentiation, respectively, under TME [25].

FAS required for proliferation is heavily based on acetyl-CoA produced from glycolysis. Inhibition of FAS-related enzymes such as SREBPs, ACC1, or downstream targets leads to defective effector T-cell responses [26]. Although modulation of SREBPs or downstream pathways is rarely attempted in cancer, they may be good therapeutic targets for the future.

FAO, a key bioenergetic pathway [27], displays a critical role in the development of tolerogenic dendritic cells (DCs) [28]. Interestingly, DCs of cancerous origin tend to accumulate oxidized lipids, thereby suppressing T-cell effector functions, which indirectly favors tumor progression [29]. It is speculated that accumulated fatty acids support FAO and therefore promote tolerogenicity in the cancer setting [30]. Furthermore, a carnitine palmitoyltransferase-1A (CPT1A) inhibitor, etomoxir, has immunomodulatory actions on $CD8^+$ T_{mem} cell differentiation [31]. However, Brenda Raud et al. have pointed out the flexible metabolic fuel choices of T_{mem} and found that CPT1A-mediated long chain fatty acid oxidation (LC-FAO) is expendable for the expansion of $CD8^+$ T cell memory [32].

PD-L1/2 in T-cell metabolism in TME

Programmed death ligand-1/2 (PD-L1/2) belongs to CD28 family, and is predominantly expressed on tumors and tumor-infiltrating myeloid cells [33, 34]. PD-L1/2 has been suspected to play a

negative role in TME by suppressing antitumor immunity by regulating inhibitory cascade on effector T cells [35, 36]. For example, a recent report has suggested that PD-1 participates in the metabolic reprogramming of activated T cells [37] by reducing Akt (protein kinase B) activation and subsequently inhibiting mammalian target rapamycin (mTOR) activity [37-39]. Indeed, reduced activation of mTOR in PD-1^{+} CD8+ T cells activates transcription factor forkhead box O1 (FoxO1), allowing the survival of exhausted CD8 T cells [39]. As glycolysis is necessary for T cells to obtain the required amount of energy for proliferation, the diversion from glycolysis to FAO likely sabotages antitumor immunity in TME. The preferential diversion from glycolysis to FAO leads to the longevity of T_{mem} cells, which possess substantial mitochondrial spare respiratory capacity (SRC) [40]. Consistently, inhibition of the PD-1 pathway in the early phases of a viral infection leads to raised mTOR signaling in virus-specific CD8+ T cells, resulting in quicker infection clearance [41]. Certainly, mTOR and Akt are key lipid regulators within the cell [42]. Similarly, inhibition of PD-1/PD-L1 leads to extensive cytotoxic T-cell infiltration into TME as reported in ex vivo, in vitro, and in vivo experimental models [43]. Blocking PD-1 in cancer patients leads to decreased tumor progression and improved survival [44, 45].

PD-1 has also been reported in T-cell exhaustion, which is counterproductive to immunity [46]. Exhaustion of PD-1–associated CD8+ T-cell existed in chronic viral infections in mice [47] and in clinical studies [48-50]. It is not surprising that, in 2014, FDA approved the first blocking antibody targeting PD-1 to treat metastatic melanoma, and up to August 2017, many drugs against the PD-1 pathway had been applied in various cancers [46]. However, a recent study has contradicted the holistic benefits of PD-1 blockade in TME as distinct responsiveness of T-cell subpopulations to PD-1 blockade was observed. The study observed that effector and central memory phenotypes were among the most affected T-cell subpopulations after PD-1 blockade but had different gene expression profiles with PD-L1 in comparison to PD-L2 [51].

T cells in TME of obese states

Although it is well established that obesity and lipid overload states are obvious causes of cancer and its progression [52-55], it has rarely been investigated how these conditions rewire the T-cell differentiation and metabolic switch in TME. Alison E Ringel et al. have recently addressed this important question and demonstrated how obesity shifts the metabolic program of TME to inhibit T-cell function and promote tumor growth. Researchers have systematically shown in a murine model that a high-fat diet (HFD) is differentially taken up by tumor cells in TME as compared to CD8+ T cells, which leads to modified fatty acid partitioning, diminished CD8$^+$ T-cell infiltration, and promotion of tumorigenesis [56]. Of course, preferential fat consumption by tumor cells makes localized T cells less efficient as fat is required to raise the number and plasticity of T cells in TME.

There are several other genes and enzymes with limited evidence but with potential to be good drug targets. The consumption of fat is not only restricted to activate the T cells, but it is also evident that under normal circumstances de novo cardiolipin synthesis keeps the function of CD8+ T cells intact. Mauro Corrado and colleagues demonstrated poor T-cell antigenic responses in T cells deficient in the cardiolipin-synthesizing enzyme—protein tyrosine phosphatase, mitochondrial-1 (PTPMT1). PTPMT1-dependent cardiolipin synthesis is also important for mitochondrial fitness, especially during T_{mem} cell differentiation or nutrition scarcity [57]. Monoacylglycerol lipase (MGL) hydrolyzes monoglycerides into glycerol and fatty acids. It is abundantly present in tumor cells, and MGL knockout (KO) mice exhibit a reduced tumor size compared with control mice. Interestingly, the reduction in tumor progression is associated with a parallel upregulation in the number of CD8$^+$ T cells. Furthermore, naïve CD8$^+$ T cells exhibit enhanced tumoricidal activity in MGL KO mice [58]. P4HA2 is a metabolism-related gene that is upregulated in cervical cancer tissues, and negatively correlates with CD8+ T cells. Knockdown of P4HA2 suppresses lipid droplet storage in cancer cells [59]. Teresa Manzo and colleagues demonstrated a progressive accumulation of long-chain fatty acids (LCFAs), which, instead of providing an energy source, hamper the mitochondrial function and rewire the lipid metabolism pathways. In addition, intrapancreatic CD8+ T cells inhibit the very long-chain acyl-CoA dehydrogenase (VLCAD) enzyme, which worsens the accumulation of LCFAs and very-long-chain fatty acids (VLCFA), subsequently

inducing lipotoxicity. In fact, recently obesity has also been described as a booster of antitumor pharmacotherapy in some cancers [60], but the mechanism remains unknown.

MDSCs in T-cell lipid (de)regulation

Myeloid-derived suppressive cells (MDSCs) manifest negative regulatory activity by promoting immunosuppression in immune-related diseases [61, 62]. In tumors, MDSCs accelerate tumor proliferation, tumor expansion, and immune escape, thereby further exacerbating the TME [63, 64]. MDSCs reshape TME by inhibiting T cells and natural killer (NKT) cells while inducing regulatory T cells (T_{regs}) and regulatory B cells (B_{regs}) [65, 66]. From recent studies, it is evident that lipid metabolism in tumor-infiltrating MDSCs (T-MDSCs) is rewired for raised fatty acid uptake, FAO upgrade, oxygen consumption rate (OCR), mitochondrial mass, and expression of core FAO enzymes [67]. It is interesting to note that only T-MDSCs, but not splenic MDSCs, raise lipid uptake [68], which implies that only infiltrating MDSCs undergo lipid metabolic reprogramming. This differential scheme of pro-tumor metabolic features sheds light on the complexity of TME.

In mammals, Liver X receptors (LXRs) are involved in lipid homeostasis. Previous studies have revealed that administration of LXR agonists initiates MDSC apoptosis and reduces tumor volume [69, 70]. In addition, lectin-type oxidized LDL receptor 1 (LOX-1) is present in PMN-MDSCs of cancer patients but is absent in healthy individuals [71]. Similarly, Caijun Wu et al. noticed the enhanced immunosuppressive role of monocytic MDSCs after administration of a multidose clinical regimen of gemcitabine (GEM). These authors have implicated that the deregulation of lipid metabolism in residual tumor cells is partially responsible for promoting immunosuppression [72]. It is plausible to conclude that tumor-derived MDSCs are forced to rewire lipid metabolism primarily because of robust lipid storage and related signaling activation.

CD36 and T-cell regulation in TME

CD36 is a scavenger receptor of oxidized lipids, and is expressed in multiple cell types, including T cells [73, 74]. Previous studies have highlighted that tumor-associated immune cells undergo CD36-oriented lipid metabolic reprogramming, which leads to immune evasion and cancer progression [75]. Shihao Xu and coworkers have reported that $CD8^+$ tumor-infiltrating lymphocytes (TILs) are responsive to lipids in the TME, mediated by CD36, which is associated with progressive T-cell dysfunction. It has been explained that T-cell dysfunction occurs in a CD36-dependent manner, which leads to a raise in oxidized low-density lipoproteins (OxLDL) in T cells, promotion of lipid peroxidation downstream, and occurrence of ferroptosis. Interestingly, overexpression of glutathione peroxidase 4 reverses lipid peroxidation to improve the effector capacity of T cells [76]. Similar evidence has been provided by other research groups, associating overexpression of CD36 with shorter survival of melanoma patients with tumor-infiltrating $CD8^+$ T cells, while CD36-depleted $CD8^+$ T cells showed greater antitumor potential and survival compared with wild-type $CD8^+$ T cells [77-80].

Importantly, there are very few identified metabolic drug targets that work in the same direction in both T_{regs} and T_{effs}. As presented above, metabolic drug targets are often context- and T-cell subtype–dependent, in which T_{regs} and T_{effs} promote or inhibit tumor, respectively. However, CD36 offers a rare opportunity because its deletion on both T_{regs} and T_{effs} results in enhanced antitumor activities [81]. For instance, Wang et al. stated that genetic knockdown of CD36 in T_{reg} cells reduced tumor growth and intratumoral T_{reg} cells, promoting the antitumor function of tumor-infiltrating lymphocytes [82]. Although only few studies have been reported in this direction, CD36 presents a viable common drug target that requires future research.

Effects of lipid metabolism on T_{regs}

The relative ratio of cytotoxic T cells and T_{regs} in TME plays a pivotal role in tumor progression and immune evasion [83]. T_{regs} contribute to immune evasion in TME [84-86]. Systematic ablation of T_{regs} in several cancer types has resulted in tumor suppression and cellular alterations within the TME [87, 88]. It is suspected that cytotoxic T_{effs} and T_{regs} follow different activation and proliferation pathways as

T_{regs} are abundant even in the unfavorable metabolic states in TME [89]. Indeed, Weinberg et al. have stressed the necessity of mitochondrial metabolism in T_{regs} to maintain their immunosuppressive function [90]. A recent study has shown raised production of FFAs by RHOA Y42-mutated gastric cancer, modulated via the PI3K pathway, which favors the accumulation of T_{regs} in a low-glucose TME. Similarly, the expression levels of FAS, CPT-1, PPARα, and PPARɣ were also higher in gastric cancer with RHOA Y42 mutation [91]. It remains unknown what metabolic switch enables T_{regs} to expand and proliferate differently from T_{effs} in the same TME. However, one study confirmed that intratumoral T_{regs} indirectly promote M2-like TAMs by boosting SREBP1-dependent lipid metabolism and then limiting the $CD8^+$ T-produced interferon-gamma (IFNγ), thereby leading to tumor progression and orchestrating tumor-associated immunosuppression [92]. In addition, inhibition of FABP5 on T_{regs} causes mitochondrial alterations characterized by impaired lipid metabolism, reduced OXPHOS, and loss of cristae structure. The authors concluded that FABP5 is a gatekeeper of mitochondrial integrity, which is necessary for normal functioning of T_{regs} [93]. An interesting observation regarding the complicity of T_{regs} and tumor cells to suppress the T-cell functioning has recently been highlighted. Xia Liu et al. pointed out that senescent T cells presented unbalanced lipid metabolism, while tumor cells and T_{reg} cells have driven increased expression of IVA phospholipase A_2, which is responsible for modified lipid metabolism and senescence observed in T cells. The inhibition of group IVA phospholipase A_2 initiated reprogramming in effector T-cell lipid metabolism, thereby stopping T-cell senescence in cancer models in vivo and in vitro [94].

Unconventional T cells in TME

γδ T cells are capable of differentiating into various subtypes of immune cells depending on the TME conditions [95]. Although the scientific knowledge on γδ T cells is underdeveloped and their proclivity as pro-tumorigenic or anticancer immune cells is still unclear [96-98], γδ T cells are potential agents against cancer cells [99]. Various studies have reported the chameleon-like nature of γδ T cells, pointing at the flexibility they exhibit in TME [100-102]. The phenomenon has been successfully

explained in squamous cell carcinoma [103] and colorectal cancer [104], where it has been suggested that TME conditions can affect the proliferation and functional nature of γδ T-cells. For example, a recent study has highlighted two distinct subtypes of γδ T cells, namely, antitumoral IFN-γ–producing γδ T cells (γδIFN cells) and IL17-producing γδ T cells (γδ17 cells) [105]. Interestingly, it has been shown that Vδ2 cells are activated, independent of MHC, by small lipid molecules, phosphoantigens (pAgs), which are derived from the mevalonate pathway [106-108]. Furthermore, Emmanuel Scotet et al. identified two different lipid-related ligands of Vγ9Vδ2 TCR in tumor cells, namely apolipoprotein A1 (Apo-A1) and ATP synthase/F1-ATPase (high-affinity apo A-I receptor). These authors revealed that Apo-A1, which is abundant in high-density lipoproteins (HDL), is needed for the activation of Vγ9Vδ2 T cells by tumors expressing F1-ATPase [109]. Similarly, a related study by Rodrigues et al. showed that Vδ2 T cells express low-density lipoprotein (LDL) receptors when they are activated and their functions can be modified once LDL attaches to their activated receptors. It has also been demonstrated that expression levels of IFN, NKG2D, and DNAM-1 are downregulated when Vγ9Vδ2 T cells are treated with LDL-cholesterol [110]. Furthermore, host-derived lipids from lung-infiltrating CD1d+B-1a cells are able to induce γδ T cells for the induction of IL-17A [111].

As a specialized type of T lymphocytes, Natural killer T cells (NKT cells) recognize lipid antigens presented through CD1d [112, 113]. NKT cells are divided into two distinct types, including I and II NKT cells, which regulate the immune response in the development and progression of tumor [114-116]. Both type I and type II NKT cells show intermodulation, but type I NKT cells are known to increase antitumor responses, while type II NKT cells are inclined towards pro-cancer activities [117], with some contextual exceptions where type I can also suppress tumor immunity [115, 118, 119]. However, tumor growth in TME is bound to consume more lipids to support its rapid proliferation and meet excessive energy needs. De novo lipid synthesis, greater and preferential fatty acid uptake from surrounding tissues in TME, and altered equilibrium of polyunsaturated fatty acids (PUFAs) and saturated fatty acids (SFAs) change the lipid repertoire of tumor cells, which can affect membrane fluidity, cell–cell interaction, and membrane protein landscape, subsequently affecting the downstream signaling cascade

[120, 121] in cancers [122, 123]. The changes in lipid repertoire are also linked to the altered structure of bio-in cancer cells [124]. In this context, HFD rich in SFA can negatively affect the capability of DCs to activate naïve T cells [125], which is critical for antitumor responses. The availability of lipids is necessary for the NKT cells development [126] as mice deficient in lysosomal lipid transfer enzyme Niemann Pick C (NPC) 2 have a decreased number of type I NKT cells [127]. There is no doubt that excess lipid states lead to the activation of type I NKT cells, which generates a proinflammatory environment in obese patients [128], while $CD1d^{-/-}$mice show reduced inflammation under similar conditions [129]. Furthermore, the antitumor potential of NKT cells in obesity is reduced and does not inhibit tumor growth [130]. However, human studies were unable to show any changes in the number of NKT cells in a hepatocellular carcinoma (HCC) model [131]. It is interesting to note that higher lipids increase the NKT cell proliferation, leading to proinflammatory responses, but an obese state reduces NKT cells, causing hindrance in tumor immunity. Further research in this direction is vital and has the potential to unveil anticancer drug targets.

Conclusion

Lipid metabolic features differ in their ability to initiate antitumor or pro-tumor responses in glucose-diminished TME infiltrated by cytotoxic T cells, T_{regs}, T_{mem}, and NKT cells (Figure 1). It may be oversimplified to argue that lipids proliferate T_{effs} and regress T_{regs}/T_{mem} in complicated TMEs where diversified forms of lipids exist. The predisposition of TME to attract and utilize excessive lipids leaves little energy for T_{effs} to expand. In a complex TME, the identification of lipid-based T-cell drug targets is context-dependent as the same genes or enzymes responsible for attracting lipids to T cells preferentially contribute to cancer cell fat intake. The type of lipid intake peculiarly affects different subsets of T cells. Formulation of a definitive hypothesis in this regard is too early. It is necessary to explore the expression difference of the same gene in different subtypes of T cells under TME to mark it as a drug target. Although CD36, SREBPs, PD-L1/2, FABP5, CPT-1, ACC1, GLUT1, and FAS has shown promising prospects to be potential metabolic drug targets of cancer, their context-dependence and varied

implications in T-cell subtypes urge for more research. Identifying the differential expression of lipid-related genes, gatekeepers, and enzymes on T-cell subsets and cancer cells that can be manipulated to draw clinical gains presents an opportunity for future research.

Figure legends

Figure 1. Differential T-cell responses after metabolic rewiring in response to lipids within TME. Raised expression of CD36 leads to T-cell dysregulation and immune evasion in TME. Excess lipids generate differential responses in T-MDSCs and S-MDSCs, leading to the activation of T_{regs} and causing T-cell dysregulation. Similarly, PD-1/PD-L1 complex converts glycolysis to FAO, thereby affecting T-cell exhaustion and playing a role in tumor progression. Under the state of metabolic competition within TME, T cells may opt to consume more lipids to compensate for depleted glucose and in-process lead to T cells activation.

TME: Tumor microenvironment; **FAO:** Fatty acid oxidation; **T-MDSCs**: Tumor MDSCs; **S-MDSCs**: Splenic MDSCs; **oxLDL**: Oxidized low-density lipoproteins; **T_{regs}**: T regulatory cells; **SREBPs**: Sterol-responsive element-binding proteins

Raised OxLDL intake: Raised intake of oxidized low-density lipoproteins (OxLDL) increases lipid peroxidation downstream and causes ferroptosis in T cells, leading to suppression of antitumor immunity.

Lipids induce metabolic reprogramming: Myeloid-derived suppressive cells (MDSCs) rewiring lipid metabolism have a direct role in remodeling TME by repressing T cells as well as natural killer (NK) cells and generating regulatory T cells (T_{regs}) and regulatory B cells (B_{regs}), supporting immune escape.

Metabolic reprogramming: As glycolysis is necessary for T cells to obtain the required amount of energy for proliferation, PD-1/PD-L1 – involved metabolic reprogramming from glycolysis to fatty acid oxidation (FAO) causes T-cell exhaustion, which is likely to sabotage antitumor immunity in TME.

Antitumor metabolic reprogramming: Sterol-responsive element-binding proteins (SREBPs) increase activated T cells with greater metabolic demands by enhancing fatty acid synthesis (FAS), therefore driving the antitumor immunity.

Lipid_Metabolic_Features_of_T

ORIGINALITY REPORT

9%

SIMILARITY INDEX

PRIMARY SOURCES

1	Yufei Wang, Anna Jia, Yujing Bi, Yuexin Wang, Guangwei Liu. "Metabolic Regulation of Myeloid-Derived Suppressor Cell Function in Cancer", Cells, 2020<br>Crossref	69 words — 1%
2	www.mdpi.com<br>Internet	59 words — 1%
3	www.frontiersin.org<br>Internet	35 words — 1%
4	Xia Yang, Wen Tang, Yongtao He, Huimin An, Jin Wang. "Development of a novel lipid metabolism-based signature to predict survival and immune response in triple negative breast cancer", Research Square Platform LLC, 2022<br>Crossref Posted Content	30 words — 1%
5	Hua Tian, Louis J. Sparvero, Tamil Selvan Anthonymuthu, Wan-Yang Sun et al. " Successive High-Resolution (H O) -GCIB and C -SIMS Imaging Integrates Multi-Omics in Different Cell Types in Breast Cancer Tissue ", Analytical Chemistry, 2021<br>Crossref	26 words — 1%
6	J. Zhao, X. Weng, S. Bagchi, C.-R. Wang. "Polyclonal type II natural killer T cells require PLZF and SAP for their development and contribute to CpG-	16 words — < 1%

mediated antitumor response", Proceedings of the National Academy of Sciences, 2014
Crossref

---

7 kuscholarworks.ku.edu — 15 words — < 1%
Internet

8 research-information.bris.ac.uk — 15 words — < 1%
Internet

9 Natural Killer T cells, 2012. — 14 words — < 1%
Crossref

10 Xia Liu, Celine L. Hartman, Lingyun Li, Carolyn J. Albert et al. "Reprogramming lipid metabolism prevents effector T cell senescence and enhances tumor immunotherapy", Science Translational Medicine, 2021 — 12 words — < 1%
Crossref

11 "Apoptosis in animal models of virus-induced disease", Nature Reviews Microbiology, 02/2009 — 10 words — < 1%
Crossref

12 www.pubfacts.com — 10 words — < 1%
Internet

13 Musso, G.. "Recent insights into hepatic lipid metabolism in non-alcoholic fatty liver disease (NAFLD)", Progress in Lipid Research, 200901 — 9 words — < 1%
Crossref

14 Xingzhe Ma, Liuling Xiao, Lintao Liu, Lingqun Ye, Pan Su, Enguang Bi, Qiang Wang, Maojie Yang, Jianfei Qian, Qing Yi. "CD36-mediated ferroptosis dampens intratumoral CD8+ T cell effector function and impairs their antitumor ability", Cell Metabolism, 2021 — 9 words — < 1%
Crossref

15 discovery.dundee.ac.uk
Internet

9 words — < 1%

16 Dieter Kabelitz. "Effector functions and control of human γδ T-cell activation", Microbes and Infection, 1999
Crossref

8 words — < 1%

17 Macho-Fernandez, Elodie, and Manfred Brigl. "The Extended Family of CD1d-Restricted NKT Cells: Sifting through a Mixed Bag of TCRs, Antigens, and Functions", Frontiers in Immunology, 2015.
Crossref

8 words — < 1%

18 Mitok, Kelly A.. "Mutation in Sortilin Identified in an Amish Population Results in Hypercholesterolemia and Insulin Resistance in Mice and Humans.", The University of Wisconsin - Madison, 2021
ProQuest

8 words — < 1%

19 hdl.handle.net
Internet

8 words — < 1%

20 www.jove.com
Internet

8 words — < 1%

21 Francis, Alyssa Victoria. "The Tumour Immune Microenvironment in Early Breast Cancer Progression.", McGill University (Canada), 2021
ProQuest

7 words — < 1%

22 Jonathan Kaye. "B and T lymphocyte attenuator regulates CD8+ T cell–intrinsic homeostasis and memory cell generation", Nature Immunology, 02/2007
Crossref

7 words — < 1%

23 Wiken, Maria. "Innate and Adaptive Immune Responses in Pulmonary Sarcoidosis.", Karolinska Institutet (Sweden), 2021 — 7 words — < 1%
ProQuest

24 Xiaohui Wang, Xiang Lin, Zihan Zheng, Bingtai Lu et al. "Host-derived lipids orchestrate pulmonary γδ T cell response to provide early protection against influenza virus infection", Nature Communications, 2021 — 7 words — < 1%
Crossref

25 Liao, Chia-Min, Michael I. Zimmer, and Chyung-Ru Wang. "The Functions of Type I and Type II Natural Killer T Cells in Inflammatory Bowel Diseases :", Inflammatory Bowel Diseases, 2013. — 6 words — < 1%
Crossref

26 Terabe, M.. "NKT cells in immunoregulation of tumor immunity: a new immunoregulatory axis", Trends in Immunology, 200711 — 6 words — < 1%
Crossref

EXCLUDE QUOTES	ON	EXCLUDE SOURCES	OFF
EXCLUDE BIBLIOGRAPHY	ON	EXCLUDE MATCHES	OFF
